# Terrestrial algae: pioneer organisms of carbonate rock solutional weathering in South China karst

**DOI:** 10.3389/fmicb.2024.1329695

**Published:** 2024-02-14

**Authors:** Ni Yan, Jiawei Zhao, Kangning Xiong, Chunliu Yang, Junqin Li, Qian Chen

**Affiliations:** ^1^School of Karst Science, Guizhou Normal University, Guiyang, China; ^2^State Engineering Technology Institute for Karst Desertification Control, Guiyang, China; ^3^College of Life Science, Guizhou Normal University, Guiyang, China

**Keywords:** carbonate rock, terrestrial algae, carbonic anhydrase activity, extracellular polysaccharide, solutional weathering

## Abstract

The formation of soil in karst ecosystem has always been a scientific problem of great concern to human beings. Algae can grow on the exposed and non-nutrition carbonate surface, inducing and accelerating weathering of rock substrates, thus promoting soil formation. Yet the actual contribution of algae to solutional weathering intensity remains unclear. In this study, we performed weathering simulation experiment on two algae species (*Klebsormidium dissectum* (F.Gay) H.Ettl & G.Gärtner and *Chlorella vulgaris* Beijerinck), which were screened from carbonated rock surfaces from a typical karst region in South China. The results showed: (1) both algae have solutional weathering effect on carbonate rock, (2) there is no difference of solutional intensity observed, yet the solutional modes are different, suggesting different ecological adaptative strategies, (3) algae on carbonate rocks have higher carbonic anhydrase activity (CAA) and secrete more extracellular polysaccharide (EPS), accelerating rock weathering. (4) The absolute dissolution amount of carbonate rock with algae participation is 3 times of that of without algae. These results indicate the significant impact of terrestrial algae on carbonate rock solutional weathering and provides quantitative evidence that terrestrial algae are pioneer species. It also contributes to our further understanding of soil formation in karst ecosystems in South China.

## 1 Introduction

Carbonate rocks are highly abundant and widely distributed throughout the world. In China, carbonate rocks cover 3.44 million km^2^, accounting for 1/3 of the land area (Li et al., 2022). Exposed carbonate rock (about 910,000 km^2^) is mainly distributed in South China, centering karst areas in Guizhou province (Yuan, 1993). The weathering of karst carbonate rocks is an important soil forming mechanism of soil resources in South China (Wang et al., 1999). Carbonate rocks mainly include limestone, chalky limestone, dolomite, and transitional rocks between them. They are composed of calcite and dolomite in varying percentages, with CaCO_3_ and CaMg(CO_3_)_2_ as the main chemical components. SiO_2_ is a minor component (Yuan et al., 2016). Based on carbonate content and the amount of acid-insoluble material, they can be broadly classified into three types: pure carbonate rocks, relatively pure carbonate rocks, and impure carbonate rocks. Although there are differences, the overall characteristics include high concentrations of Ca and Mg, and the absence or only trace amounts of elements such as Si, Al, and Fe. The combined content of Al_2_O_3_ and Fe_2_O_3_ in carbonate rocks is less than 1%. Another characteristic is that soluble minerals such as calcite and dolomite constitute over 90% of the mineral composition, even reaching 99%, while the content of insoluble material is relatively low. Over 90% of the material in carbonate rocks is dissolved and transported away through weathering and erosion processes, making it challenging for a significant proportion to remain as soil. Therefore, relying solely on pure carbonate rocks makes it difficult to weather a substantial amount of soil particles (Lian, 2010). Williams (1957) pointed out that the soil formation begins at the moment organisms (green plants, animals and microbes) start their development in rock crevices. The reproduction of the organisms on the surface of rocks and the ceaseless weathering process initiates the formation of soil particles, which eventually evolve to soil. Williams’ research has not yet identified the pioneer organisms of pedogenesis, which involves weathering of parent rock to form parent material and the process by which parent material forms soil. In searching for pioneer organisms, researchers in the area of biokarst study have started rigorous discussions about fungi, algae, lichens, and other organisms living in karst environment, mainly focusing on the weathering process and the roles they play in this process (Danin and Gerson, 1983; Cao et al., 1993; Katja, 2000; Smith et al., 2000; McIlroy de la Rosa et al., 2014; Cao et al., 2020).

The formation of karst landforms in South China began after the sea regression. The rapid development of karst landforms resulted from the east Asian monsoon climate in tandem with the collision of the Indian plate and the Eurasian plate in the late Eocene which led to the uplift of the Qianhai-Tibet plateau (Sweeting, 1986; Liu et al., 1993). The evolutionary history of algae suggests, that it was likely the earliest pioneer organism which colonized on bare rock. Yan et al. (2021) preliminarily demonstrated the co-evolution of algae and karst landform in the study of algae diversity in karst. Algae were the earliest photosynthetic autotrophic life forms in nature, with a simple structure, tremendous kinds, great abundance, and a wide distribution. Algae had existed throughout carbonate rock evolution for thousands (or maybe millions) of years. From marine deposition (aquatic) to land exposure (terrestrial), the algae had adapted to the terrestrial environment via a series of adaptation mechanisms that ensured their survival, not only simply to live, but also to initiate changes through their metabolic process, thus activating the carbonate lithosphere (Cao et al., 2001). This is not only related to the regulation of atmospheric CO_2_ (Burlacot et al., 2021; Zhang M. et al., 2022), but also to soil formation. The soil formation mechanism of karst is the current research focus (Li et al., 2020; Zhu et al., 2021; Zhang S. R. et al., 2022) and it is an inevitable problem in the discussion of restoration strategies for karst degraded ecosystems (Wang et al., 2022; Xiao and Xiong, 2022).

Folk et al. (1971) studied the rock dissolution effect of algae and proposed the term “Phytokarst.” Other researchers focused their attention on surface morphology. For example, Danin and Gerson (1983) found that the algae activity formed irregular corrosion marks on the macro level, while on the micro level, it formed a spongy or honeycomb drilling surface. Jennings (1985) pointed out that whether the surface of limestone was covered by vegetation (including algae, lichens, mosses and higher plants) is an important factor affecting limestone dissolution. Zhang (1993) demonstrated that algae dissolution was a common phenomenon in karst areas and algal cover has a positive effect on karst erosion. Tian et al. (2002, 2004) found that algae can loosen the shallow rock surface by 0 ~ 2 cm through adhesion, penetration, filling and drilling, thus reducing the hardness of rock surface and making it rough. Recently, domestic and foreign scholars focused their attention on the dissolution effect of algae metabolites and active substances secreted in their life process. The main contents of mucus and remains of algae are polysaccharides (Flemming et al., 2007; Kehr and Dittmann, 2015), which mediate the adhesion to the rock surface, complex minerals (Seiffert et al., 2014; Ng Daphne et al., 2016), and being further oxidized into oxalic acid or butyric acid through the action of microorganisms (Sun et al., 2014), thus strengthening the dissolution of carbonate. While studying the dissolution abilities of blue-green algae and their physiological activity, Nie et al. (2012) found that the dissolution rate of limestone with blue-green algae was much faster than that of bare rocks and related it to the activity of carbonic anhydrase (CA). Xie and Wu (2014a) carried out quantitative research on utilization of calcium in calcium carbonate and found that algae mainly used dissolved inorganic carbon (DIC) formed in the process of calcium carbonate dissolution, suggesting that the concentration of CO_2_ in atmosphere and extracellular CA are important factors affecting calcium carbonate dissolution. The widespread metal enzyme CA with zinc as the active center can catalyze the mutual conversion of CO_2_ and HCO_3_^−^. It can catalyze the hydration reaction of CO_2_ to generate H^+^, thus destroying the ionization balance of CaCO_3_, and driving the dissolution of calcium carbonate in limestone karst areas (Wu et al., 2015). In their study about CO_2_ concentration gradient effect on CA activity, Huang et al. (2018) indicate that Ca^2+^ plays a non-negligible role when studying the relationship between CA and dissolution dynamics. The research of Gaylarde and Little (2022) pointed out that algae accelerate the carbonate rock weathering through enzyme secretion, thus speeding up the karst carbon soil formation.

The above research results were mainly from qualitative observations from inland karst and coastal carbonate rock, the same outcomes were also found in inland and coastal environments. Yet the dissolution phenomena were not related to specified species, nor does it address the possible dissolution effect caused by the algal ecotype difference. The inland algae were simply categorized into 3 groups (i.e., aerial, aquatic, and soil groups), which caused confusion and unclear research directions. There are differences between the ecological adaptation strategies of different ecotypes of algae or different subgroups of the same ecotype, which leads to the differences of the structure, function, and contents of metabolites, thus affecting the physical and chemical properties (such as pH values, conductivity, and HCO_3_^−^ level) and in turn affecting the intensity and rate of dissolution. Nie et al. (2012) pointed out that the dissolution rate of blue-green algae living on the shadow side was significantly higher than that of the blue-green algae living on light-exposure side due to the significantly higher CA activity of the living algae on the shadow side. Rui and Yi (2016) comprehensively discussed the composition, function, and structure of the exopolysaccharides (EPS) of different algae and showed that there are differences among species of same ecotype. The previous studies about the mechanism of dissolution, although focused on the active substances secreted by the algae, lack of consideration of the evolutionary history and related habitat. Therefore, in this study, we tried to bring insight from aspects of aquatic-terrestrial systematic evolution via performing an indoor simulation experiment on the aquatic and terrestrial algae species screened from the karst center in Huajiang research area, Guanling-Zhenfeng, Guizhou, China. This is the perfect experiment location due to its history of sea immersion and regression. Additionally, in this location there is great amount of bare carbonate rocks; the algae cover can be clearly seen in the deep recessions and crevices on rock surface; and some of the rock gaps, crevices, or recessions still have a small amount of soil left, which kept the soil structure and relatively high nutrition level (Li et al., 2006). These phenomena lead to a quite meaningful scientific questions: after natural rainfall, what is the carbonate rock dissolution process and law in the short-term ponding on the completely soilless rock surface? and does the intensification of dissolution cause the accumulation of former soil continuum?

Thus, in this study, we used a carbonate rock slice that was ground from the original bedrock to construct an algae-rock-water environment simulating the after-rainfall bare rock recession microenvironment in liquid cultures of *Chlorella vulgaris* Beijerinck and *Klebsormidium dissectum* (F.Gay) H.Ettl & G.Gärtner. Our objective is to investigate the potential dissolution mechanisms of algae during the karst ecological succession process in the weathering of exposed carbonate rocks. We expect to elucidate the relationship between pioneer species and soil formation from the perspective of algal system evolution, provide empirical evidence for soil formation and accumulation in the karst regions of South China.

## 2 Materials and methods

### 2.1 Overview of the research area

**Figure 1:**
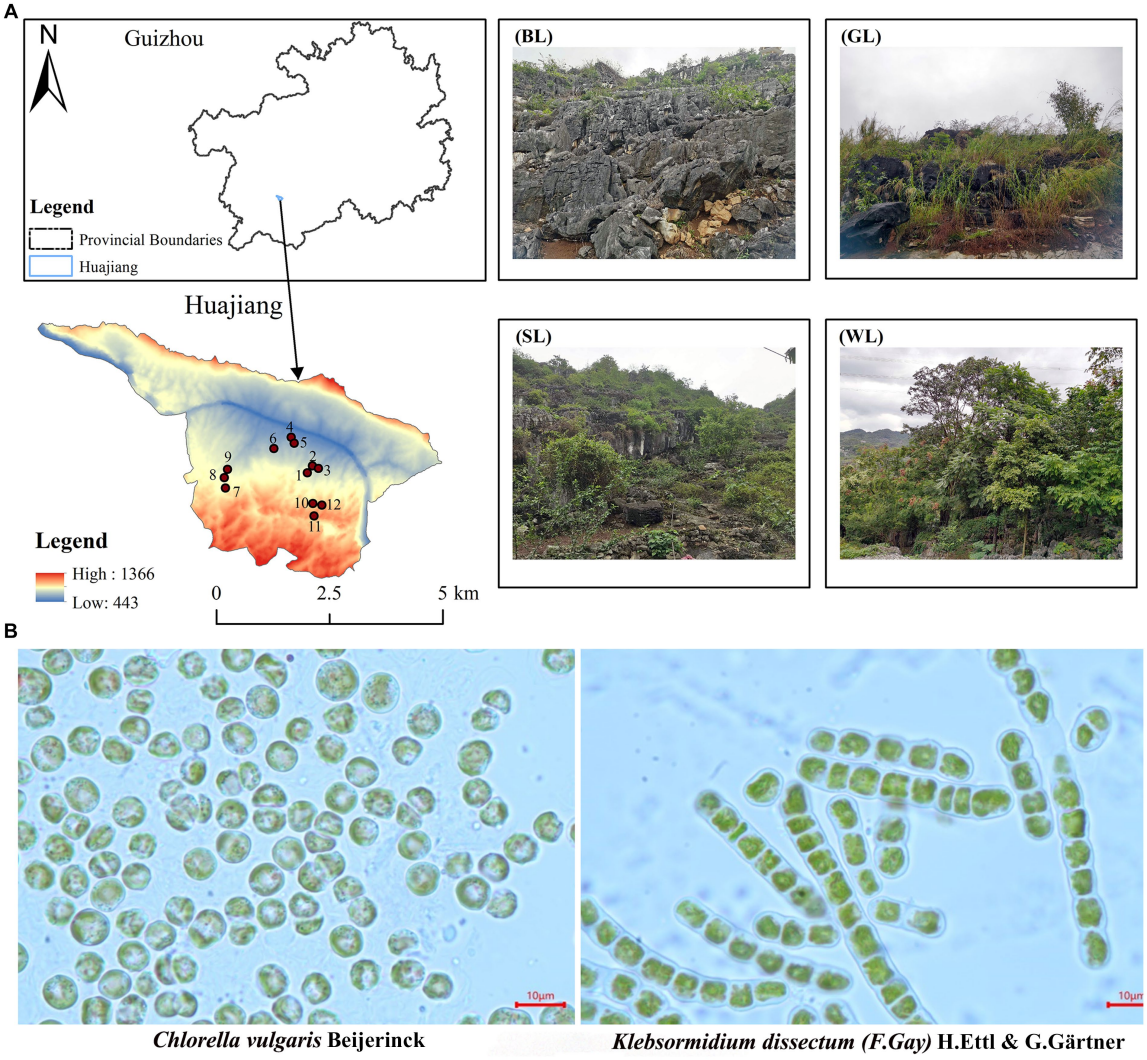
Sample area and quadrats setting **(A)** and algae species **(B)** separation from carbonate rock surface in Huajiang karst research area (BL, bare land; GL, grass land; SL, shrub land; WL, wood land).

The research area is located at the border region between Guanling County and Zhenfeng County, Guizhou Province, South China (105°36′30″-105°46′30″E, 25°39′13″-25°41′00″N), where is the typical karst area of South China (Xiong et al., 2016; Figure 1). The karst landform in the research area was well-developed, accounting for 87.92% of the full area. The geological composition is mainly carbonate rocks with a high exposure rate of bare bedrock. The potential, mild, moderate, and severe rocky desertification accounted for 24.54%, 40.48%, 17.93%, and 17.06%, respectively (Xiong et al., 2011). The exposed Devonian, Permian, Triassic, Jurassic, Cretaceous, Tertiary and Quaternary strata (Chen, 1984) spanned the geological history of more than 400 million years from the Paleozoic, Mesozoic to Cenozoic. This is an optimal research area for algae sampling (Chen et al., 2023) and carbonate rock weathering and dissolution, because it has witnessed and recorded the aquatic and terrestrial plant evolution. This area is also a treasure trove of algae fossils at different strata, which can be excavated and studied. Overall, this study aids in our understanding of the continuous geological changes and the formation of landscapes in the area.

### 2.2 Material

Algae species: the experimental algae species were collected from the fresh samples on carbonate rock surfaces in Huajiang, a typical karst research area in South China. The total area of the sampling region was 51.62 km^2^, including bare land (BL), grass land (GL), shrub land (SL) and woodland (WL). The fresh samples were cultured, separated, and purified in lab (Jafarpoor et al., 2022). Two dominant algae species of different forms (*Chlorella vulgaris* Beijerinck and *Klebsormidium dissectum* (F.Gay) H.Ettl & G.Gärtner, which belong to *Chlorella* Beijerinck, Chlorellaoideae, Charophyta and *Klebsormidium,* Klebsormidiophyceae, respectively) were screened out and used after expansion (Figure 1). *Chlorella vulgaris* distribute on the bare rock surface of the above four habitats, and *K. dissectum* distribute on the bare rock surface of wood land. The two purified algal strains came from bare rocks in the WL.

Besides being widely distributed in our study area, the two species can colonize the surface of carbonate rocks much faster than other algae we isolated in the same area (In preparation).

Algae culture: for experiments, algal cells were first cultured to a stationary stage on a shaker at (26 ± 1)°C and 2000lux in light period 12 h/12 h using BG11 (Na&N 1500 mg/L, MgSO_4_ 36.6 mg/L, C₆H₈O₇ 6 mg/L, C₆H₈FeNO₇ 6 mg/L, H_3_BO_3_ 2.86 mg/L, ZnSO_4_·7H_2_O 0.22 mg/L, CuSO_4_·5H_2_O 0.08 mg/L, K_2_HPO_4_ 30.5 mg/L, CaCl_2_ 36 mg/L, EDTANa_2_ 1 mg/L, Na_2_CO_3_ 20 mg/L, MnCl_2_·4H_2_O 1.81 mg/L, Na_2_MoO_4_·2H_2_O 0.39 mg/L, CoCl_2_·8H_2_O 0.0409 mg/L). Before the dissolution experiment, the freshly grown cell suspension (at the stationary stage) was centrifuged (4,000 r/min, 5 min), rinsed several times with 0.1 mol/L oxalate-EDTANa_2_, and finally washed several times with sterile distilled water to remove surface adsorbed metals and extracellular metabolites as much as possible.

Carbonate rock slices (CRSs): while collecting algae samples, 20 kg carbonate rocks covered by algae were also collected. The rocks were washed to remove the algae and made into similar round slices with 1 cm in diameter and 2 mm in thickness. The round slices were finely polished, washed by ultra-pure water, dried, and weighted for standby. The main contents of the rock samples are Ca: 357.49 g/kg, Mg: 2.45 g/kg, Fe: 2.64 g, Mn: 0.08 g/kg.

BG11 calcium-deficient liquid medium: 16 g of BG11 calcium deficient medium dry powder (CaCl_2_ 0 mg/L) were weighted and diluted to 1 L with distilled water. The pH value was adjusted to 7 ~ 8 with NaOH. The liquid medium was sealed and placed in the autoclave for sterilization at 121°C for 30 min, and then placed in the sterile operation table to room temperature for standby.

### 2.3 Method

#### 2.3.1 Simulation experiment

We set up experimental groups, including *C. vulgaris* and carbonate rock slice group (CS) and *K. dissectum* and carbonate rock slice group (KS), *C. vulgaris* group (CH), *K. dissectum* group (KL) and control group only contained carbonate rock slice (ST).

To simulate a natural pond environment with bare rock surface carrying algae, we performed a controlled experiment of culturing algae with carbonate rock slices to construct an “algae-rock-water” microenvironment. Briefly, 110 mL of sterilized culture medium (BG11 calcium-deficient liguid medium) and one piece of carbonate rock slices was added to a 150 mL triangular flask. 1 mL of *C. vulgaris* (CS) or *K. dissectum* (KS) grinds fluid was added to the above triangular flasks, and equal amount of sterile water was added to the control (ST). In addition, we set up a control group to which only *C. vulgaris* (CH) or *K. dissectum* (KL) was added to clarify the effect on the culture solution after solubilization of carbonate rock slices. For all treatment, the initial inoculum amount of *K. dissectum* was 0.236 mgwet·L^−1^ and the initial inoculum amount of *C. vulgaris* was 0.884 mgwet·L^−1^. Three replicates were set for each group of tests, and simulations were conducted under the same culture conditions. According to the microenvironment condition of the sampling day (04/12/2021), the cultivation environment factors were set as light intensity: 2000Lux; light period: 12 h/12; temperature: (26 ± 1)°C; relative humidity: 60–70%. On the 0th, 5th, 10th, 15th, 20th and 25th day of cultivation, 18 mL/bottle of the sample fluid were collected under sterile condition. Carbonic Anhydrase Activity (CAA), Exopolysaccharides (EPS), Potential of Hydrogen (pH), Electrical Conductivity (EC), Ion Concentration (Ca^2+^, Mg^2+^, Fe^2+^, and Mn^2+^), Algae Biomass (AB), and Content of Chlorophy a (Chl-a) were then measured.

#### 2.3.2 Measurement methods

(1) Carbonic Anhydrase Activity (CAA): We took three portions of the sample solution (0.5 mL each) and added 5 mL of barbital buffer solution (20 mmol·L^−1^, pH = 8.3) to each sample and mix them evenly. We used the centrifuge at 16000r·min^−1^ for 15 min, collected the supernatant, and then stored at 4°C for testing. To determine CAA we carried out the pH meter method. We added 0.5 mL of the supernatant to 10 mL of barbital-KOH buffer (pH = 8.3), and stirred it evenly. We then placed the pH meter in the reaction solution in a beaker, recorded the initial pH value, and quickly added 4.5 mL of frozen saturated CO_2_ aqueous solution until the pH dropped by one unit (such as 8.30 to 7.30). Once that occurred we stopped timing, recorded the reaction time, and used the same sample as the control after boiling. CAA in the sample solution is expressed by the number of enzyme activity units per mL (U·mL^−1^). The calculation formula of enzyme activity unit is: U = 10[(T_0_/T_1_)], T_0_ is the time of pH change after adding boiling leaching solution, and T_1_ is the time of pH change after adding unboiled leaching solution (Zhang et al., 2017).
(2) Exopolysaccharides (EPS): To determine polysaccharides we utilized the phenol-sulfuric acid method. In this method, polysaccharides are hydrolyzed into monosaccharides through the action of concentrated sulfuric acid, and then rapidly dehydrated to form aldehyde derivatives, and then condensed with phenol or anthrone to form orange compounds. The absorbance value of this compound at 490 nm wavelength is linearly dependent with its mass concentration, thus its content can be determined by photoelectric colorimetry. We put 0.0 mL, 0.2 mL, 0.4 mL, 0.6 mL, 0.8 mL, 1.0 mL, 1.2 mL, 1.4 mL, 1.6 mL, and 1.8 mL of the 40 μg·mL^−1^ glucose standard solution into tubes with stoppers, and then added distilled water supplementing the solution to 2 mL. We shook the solution in each tube well and then added 6 mL of prepared phenol-sulfuric acid solution (1 mL of 6% phenol solution and 5 mL of concentrated sulfuric acid, mixed by vortex oscillation) to each tube. Once again, we shook the tubes well and then let the contents bath in 90°C water for 20 min. We made the first tube the blank control, and measured the absorbance at 490 nm, thus establishing the glucose standard curve. Next, we took 1 mL of sample solution, added distilled water to make up to 2 mL, and then added 6 mL of prepared sulfuric acid-phenol solution. We shook this solution well and then let it bath in 90°C water for 20 min. We measured the absorbance value at 490 nm, and calculate the sample concentration (Tang, 2015).
(3) Potential of Hydrogen (pH), Electrical Conductivity (EC): We determined pH and EC by using a desktop pH meter (pH100) and conductivity meter (DDBJ-350).
(4) Ion Concentration: We filtered the culture solution through a 0.45 μm filter membrane and nitrated it with ultrapure 2% nitric acid, and finally used plasma emission spectroscopy (ICP-OES) to determine the concentrations of Ca^2+^, Mg^2+^, Fe^2+^, and Mn^2+^.
(5) Algae Biomass (AB): To determine the AB we used the optimal density method. We added 2.5 mL of the sample solution into a small beaker. After shaking the beaker well, we took an appropriate amount into a glass colorimetric dish, and measured it at 560 nm with a 752 N UV–VIS spectrophotometer. The OD560 value can be considered as an approximation of the algae cell AB (Cheng et al., 2012).
(6) Chlorophy a (Chl-a): To measure the algae Chl-a, we used the ethanol extraction method. We took 5 mL of mixed algae solution and placed it in the centrifuge at 4000r·min^−1^ (4°C) for 10 min. We discarded the supernatant and added 5 mL of 95% ethanol into the centrifuge tube, making sure to avoid light for 12 h in a refrigerator at 4°C. We used the centrifuge once again at 4000r·min^−1^ (4°C) for 10 min. We took the supernatant, fixed the volume to 5 mL, and then used 95% ethanol solution as blank contrast. We then measured the absorbance values at the wavelength of 665 nm and 649 nm, respectively. We calculated Chl-a (mg·L^−1^) according the formula (Zhang et al., 2017): Chl-a (mg·L^−1^) = 12.7 × A665-5.76 × A649.

### 2.4 Mathematical analysis and statistical methods

#### 2.4.1 Annual absolute dissolution

In this study, we used the standard dissolution test piece method to calculate the annual absolute dissolution amount (Yuan and Cai, 1988). The calculation formula is as follows:

$ER=\left(W_{1}-W_{2}\right)\times 1,000\times T\times 365^{-1}\times S^{-1}$

ER is the annual dissolution amount per unit area, in mg·(cm^2^·a)^−1^; W_1_ is the initial weight of the test piece (g); W_2_ is the weight of the test piece after placement (g); T is the storage time (d); S is the surface area of the test piece (about 2.199 cm^2^).

#### 2.4.2 Data and diagram analysis

**Figure 2:**
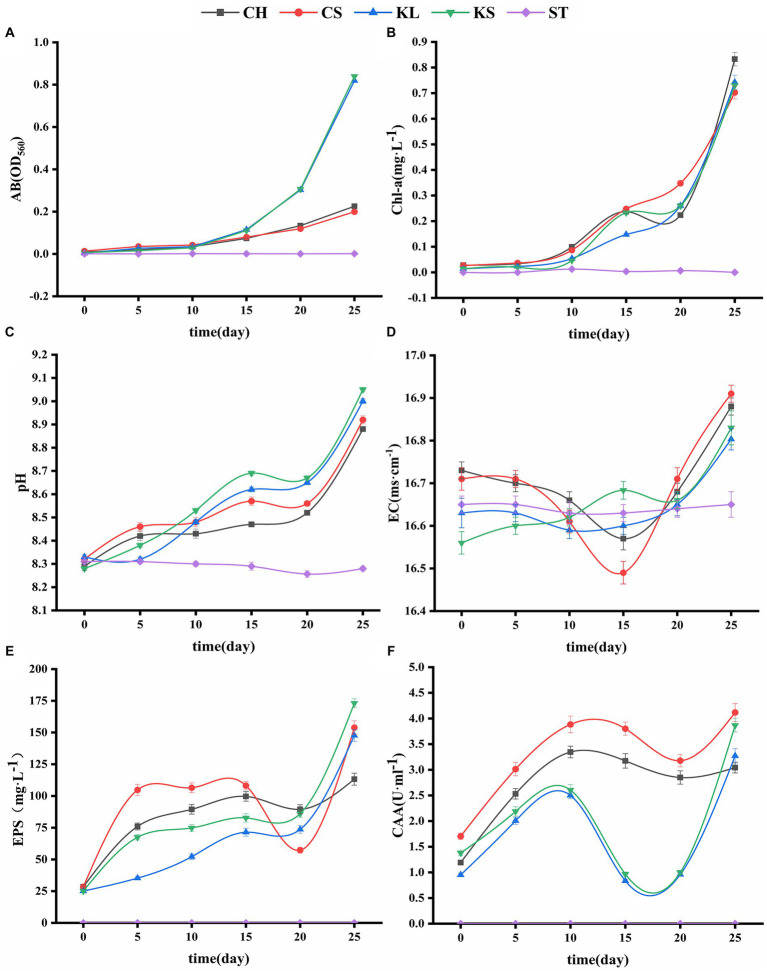
Variations curve of algae biomass (AB) **(A)**, chlorophyll a (Chl-a) **(B)**, potential of hydrogen (pH) **(C)**, electrical conductivity (EC) **(D)**, exopolysaccharides (EPS) **(E)**, carbonic anhydrase activity (CAA) **(F)** with time. *C. vulgaris* and carbonate rock slice group (CS), *K. dissectum* and carbonate rock slice group (KS), *C. vulgaris* group (CH), *K. dissectum* group (KL), control group only contained carbonate rock slice (ST).

**Figure 3:**
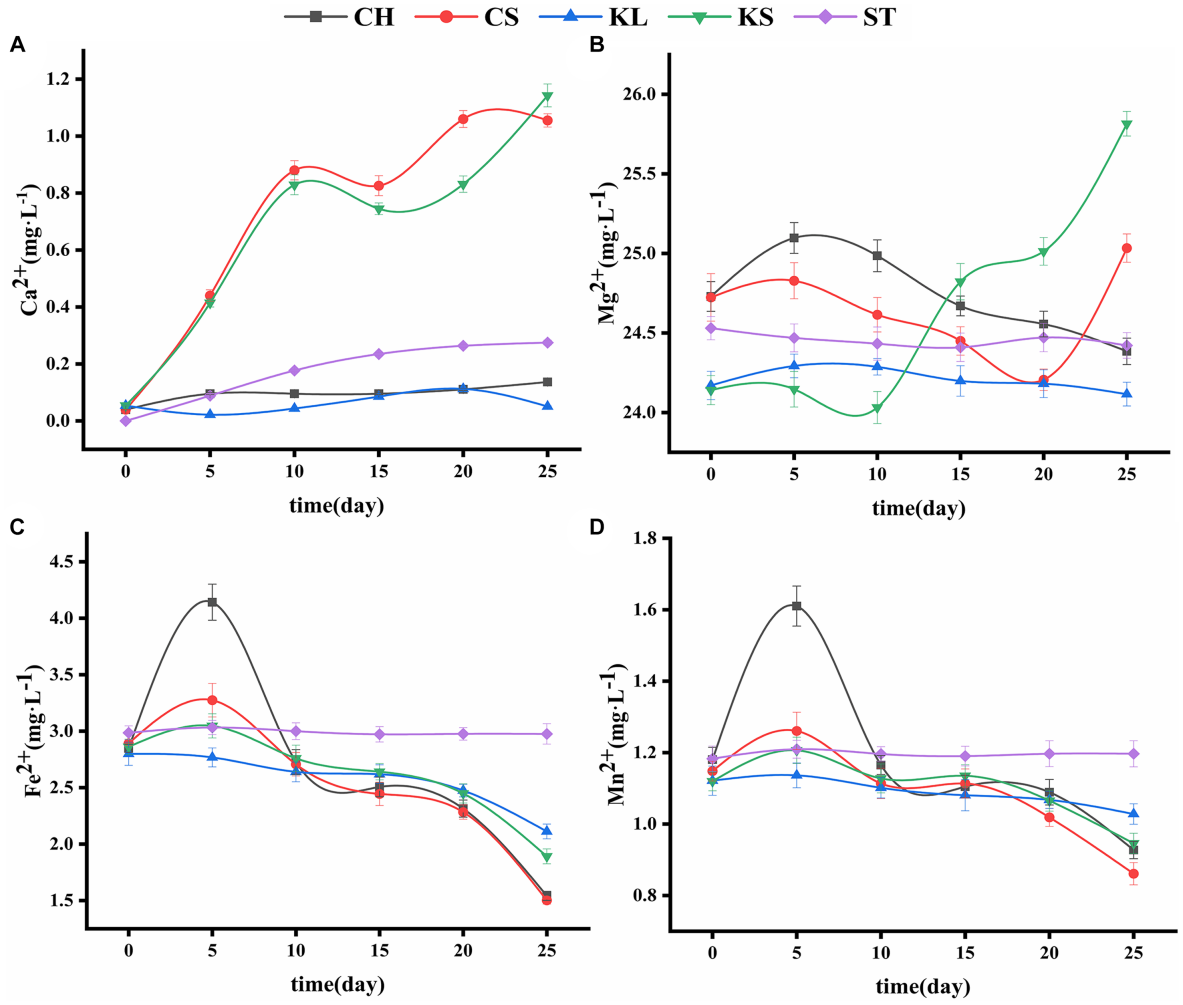
Variations curve of Ca^2+^ **(A)**, Mg2^2+^ **(B)**, Fe^2+^ **(C)**, Mn^2+^ **(D)** with time. *C. vulgaris* and carbonate rock slice group (CS), *K. dissectum* and carbonate rock slice group (KS), *C. vulgaris* group (CH), *K. dissectum* group (KL), control group only contained carbonate rock slice (ST).

**Figure 4:**
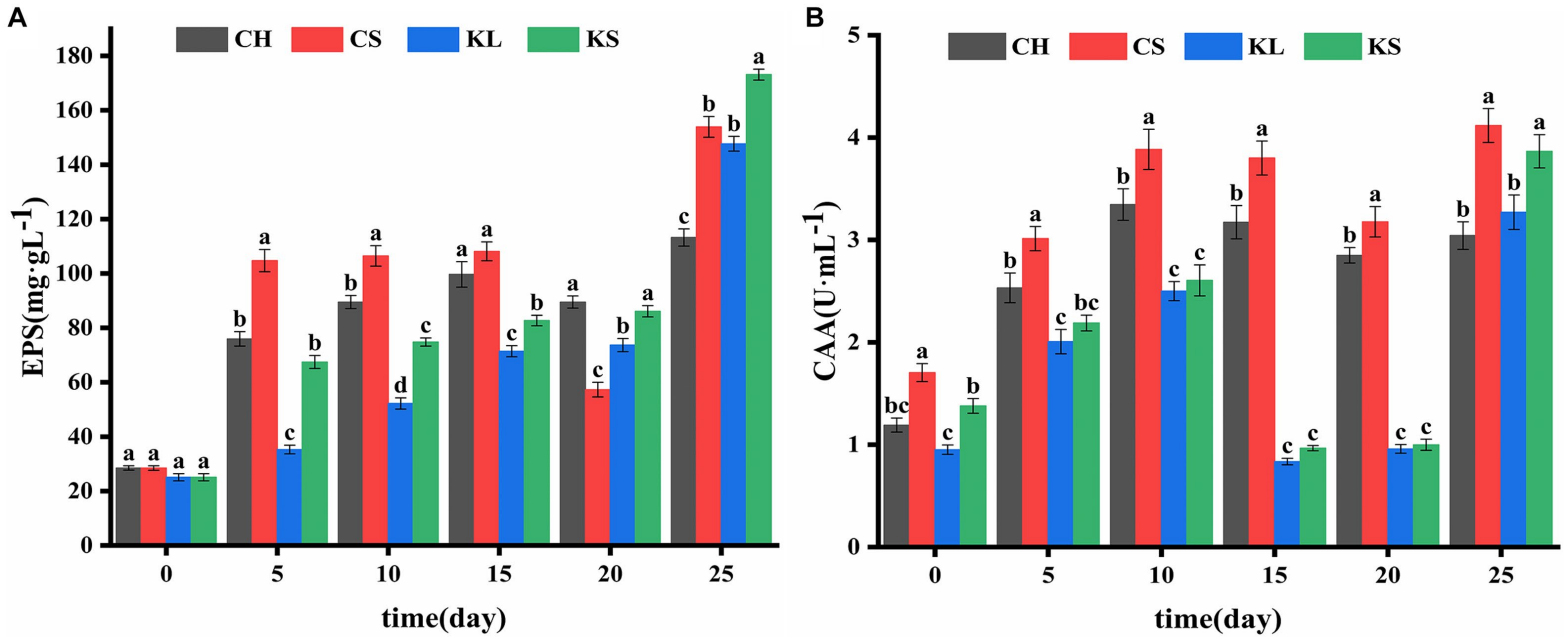
Comparison of exopolysaccharides (EPS) **(A)** and carbonic anhydrase activity (CAA) **(B)** between *Chlorella vulgaris* and *Klebsormidium dissectum*. *C. vulgaris* and carbonate rock slice group (CS), *K. dissectum* and carbonate rock slice group (KS), *C. vulgaris* group (CH), *K. dissectum* group (KL).

**Figure 5:**
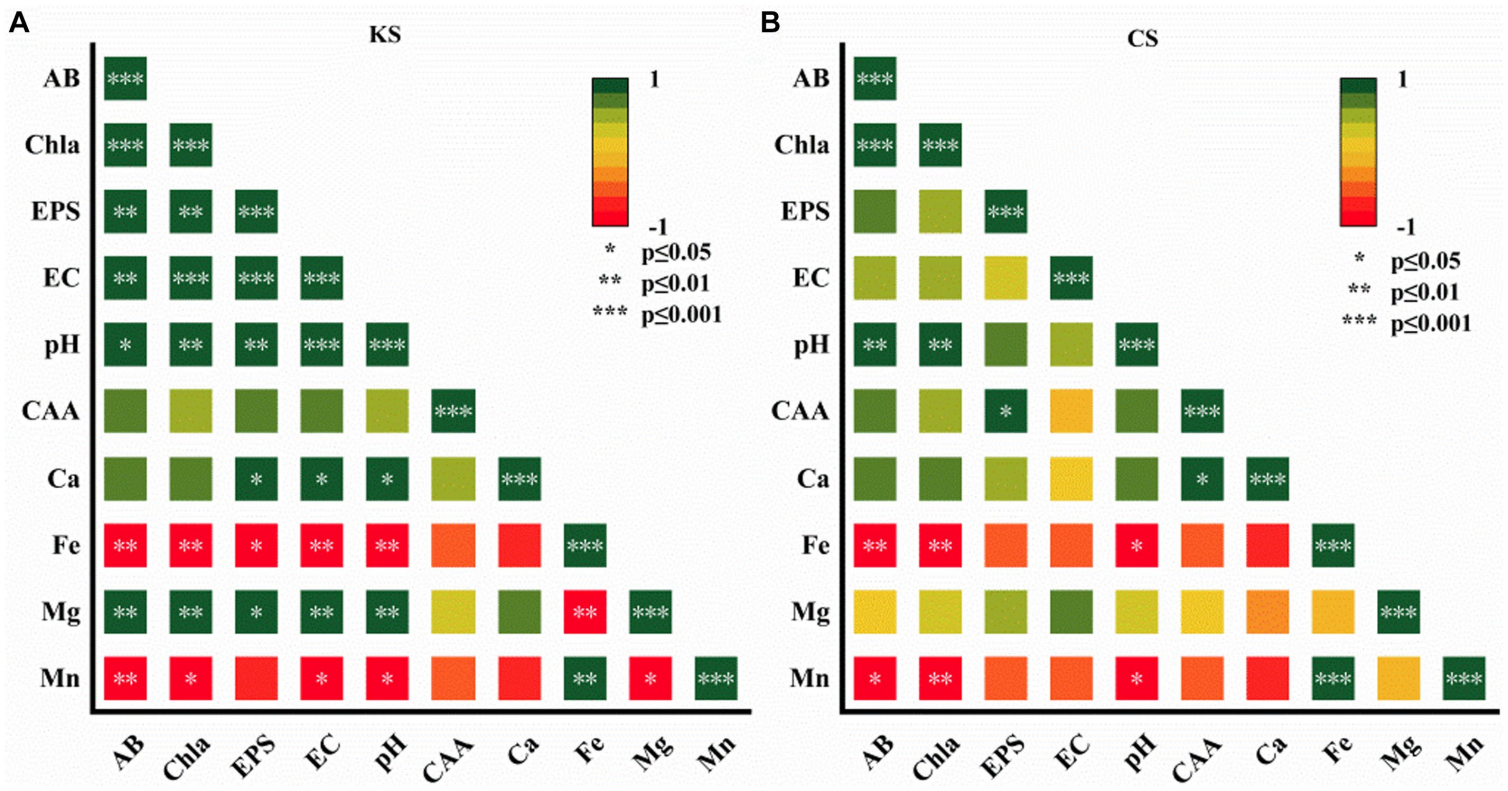
Pearson correlation analysis of algae biomass (AB), content of chlorophy a (Chl-a), exopolysaccharides (EPS), electrical conductivity (EC), carbonic anhydrase activity (CAA), potential of hydrogen (pH), ion concentration (Ca^2+^, Fe^2+^, Mg^2+^, Mn^2+^) for *K. dissectum* and carbonate rock slice group (KS) **(A)**, and *C. vulgaris* and carbonate rock slice group (CS) **(B)**.

SPSS 22, ORIGIN 21 and other software were used to sort out the data and perform the Pearson correlation analysis on CAA, EPS, pH, EC, Ion concentration (Ca^2+^, Mg^2+^, Fe^2+^ and Mn^2+^), AB, and Chl-a. The broken line chart (Figures 2, 3), bar chart (Figure 4) and correlation heat map (Figure 5) were depicted to reflect the variation law of each measured component with time.

## 3 Results

### 3.1 Algae biomass (AB) and chlorophyll a (Chl-a)

The AB and Chl-a content of the four groups (CS, KS, CH, and KL) with the algae culture increased with the number of culturing days (Figures 2A,B). The ST group stayed unchanged, since no algae was injected. The AB content increased slowly during the first 10 days in the CS, KS, CH, and KL groups. After 10 days, the 2 groups of *K. dissectum* (KS and KL) quickly increased. The increasing rate of the *Klebsormidium* groups was significantly higher than that of the *Chlorella* groups, although the initial content of Chl-a in the *Chlorella* group (0.884mgwet·L^−1^) was significantly higher than that of *Klebsormidium* groups (0.236mgwet·L^−1^). The AB and Chl-a contents of CS and KS groups were slightly higher than that of the CH and KL groups.

### 3.2 Potential of hydrogen (pH) and electrical conductivity (EC)

The pH variation of the shaking flask experiment of the two types of algae were shown in Figure 2C. The pH values of the CS, KS, CH, and KL groups continuously increased with culturing days. At the 25th day, the pH value of the CS group increased from 8.32 (0 day) to 9.05 (7.21%), and that of KS group increased from 8.28 (0 day) to 9.05 (9.30%). Within 25 days, the pH values of the CH and KL groups were lower than that of the CS and KS groups. The pH value of the ST group varied from 8.26 to 8.31. This result was likely due to the dissolution of CO_2_ in air, forming H_2_CO_3._

The EC values of the ST group were in the range of 16.62 ~ 16.65 ms·cm^−1^, and thus relatively stable during the experiment period. The EC values of the CH, CS, KL, and KS groups fluctuated greatly. The EC values of CH, CS, and KL had a similar trend: descending first and then ascending. Whereas the EC values of the KS group kept ascending (Figure 2D). The EC fluctuation range of the two groups CS, KS were higher than that of CH and KL groups.

### 3.3 Variations of the contents of Ca^2+^, Mg^2+^, Fe^2+^, and Mn^2+^

The changes in the Ca^2+^ concentration of the two types of algae were shown in Figure 3A. The slight change of Ca^2+^ concentration in calcium-deficient medium (i.e., the concentration of Ca^2+^ was 0 mg·L^−1^) in the CH and KL groups indicates the very limited Ca^2+^ release of the algae. The amount of Ca^2+^ in the ST culture was also quite small, due to the solution action on the carbonate rock slices. Comparing with ST, CH, and KL groups, the Ca^2+^ contents in CS and KS groups showed an obvious increasing trend, with increases from 0.0396 mg·L^−1^ to 1.0552 mg·L^−1^, and from 0.0539 mg·L^−1^ to 1.1429 mg·L^−1^, respectively. The carbonate rock piece is the only source of Ca^2+^ in calcium-deficient medium, thus the increase of Ca^2+^ in the CS and KS groups indicates the dissolution effect of the two algae on rock.

The concentration of Mg^2+^ in ST group was relatively stable (24.4557 ± 0.0443 mg·L^−1^) (Figure 3B), while that of the KL and CH groups shown a decreasing trend (from 25.0968 mg·L^−1^ and 24.2928 mg·L^−1^ to 24.3844 mg·L^−1^ and 24.1152 mg·L^−1^, respectively). The concentration of Mg^2+^ in the medium decreased due to the absorption of the algae. However, the corresponding results in KS and CS groups shown that the content of Mg^2+^ was not continuously decreasing. Rather, it rose back rapidly at the 10th and 20th day, reflecting the dissolution of the carbonate rock slice. The dissolution amount of KS was higher than that of CS.

The variations of Fe^2+^and Mn^2+^ were consistent with each other (Figures 3C,D). The contents of Fe^2+^ and Mn^2+^ in CH, CS, KL, and KS groups increased first and then continued to decrease. The increase that occurred during 0d-5d was likely due to the cell discharge or the bring-in at the inoculation. The decrease that occurred after 5d reflects the consumption of the two types of ions in the culture solution by algae. The final concentrations of the Fe^2+^ and Mn^2+^ in the CH, CS, KL, and KS groups were lower than that of the ST group (2.9901 ± 0.023 mg·L^−1^), indicating that the two kinds of algae have no direct dissolution effect on carbonate rock slices for Fe^2+^ and Mn^2+^.

### 3.4 Exopolysaccharides (EPS) and carbonic anhydrase activity (CAA)

The results of the determination of exopolysaccharide (EPS) of the two algae species (Figure 2E) showed that the EPS secretion of the KS and KL groups increased gradually with time. The increase was slow during 0d-20d, with the content less than 100 mg·L^−1^. After 20d, the EPS content increased rapidly, and the yield reached 173 mg·L^−1^ by the 25d, which was 7 times the initial amount. The EPS secretion amounts of CH and CS groups were significantly higher than that of *K. dissectum* (*p* < 0.05) in the first 15d (Figure 4A). From 15d-20d, the secretion amount slightly decreased and the rose up again, reaching 154 mg·L^−1^ at the 25d, which was 5 times the initial amount. Comparing the two kinds of algae, there was no difference of EPS secretion amount between them at the 0d (*p* > 0.05). In the first 15 days, the secretion amount of *C. vulgaris* was significantly higher than that of the *K. dissectum*. Eventually, the secretion amount of *K. dissectum* surpassed that of the *C. vulgaris* (*p* < 0.05). Within each group, the EPS contents was CS > CH, KS > KL (p < 0.05), i.e., the two kinds of algae cultured with carbonate rock slices secreted more EPS.

The measured CAA mainly comes from the extracellular CA secreted by algae during the growth process. As shown in Figure 2F, the two algae showed similar variation patterns: the activity rapidly increased in the early stage, slightly decreased in the middle stage, and recovered in the later stage. In the first 20 days, the CAA of *C. vulgaris* was significantly higher than that of *K. dissectum* (*p* < 0.05) (Figure 4B). By the 25d, the CAA of KL and KS rapidly increased, reaching a similar value to that of CH and CS, and thus no significant difference was observed (*p* > 0.05). Within each group, the CAA of CS was significantly higher than that of CH (*p* < 0.05) and the CAA of KS was higher than that of KL, yet there was no significance until 25d (*p* < 0.05). Generally, the CAA of the two kinds of algae showed as: experimental group > control group.

### 3.5 Pearson correlation analysis

As shown in Figure 5, for the KS group, Ca^2+^/ Mg^2+^, pH, EC, and EPS were positively significantly correlated. Ca^2+^/ Mg^2+^ and CAA were positively correlated, although not significantly. Fe^2+^/Mn^2+^ were negatively significantly correlated to AB, Chl-a, pH, EC, EPS. For the CS group, Ca^2+^ was positively significantly correlated to CAA, and positively correlated to pH and EPS, although not significantly. Fe^2+^/Mn^2+^ were negatively significantly correlated to pH, AB, and Chl-a. The pH of both groups was positively significantly correlated to AB and Chl-a. Correlation analysis further indicated the function of algae on carbonate rock weathering dissolution, which varied with algae species.

### 3.6 Absolute dissolution amount

Under the simulation condition that is used in current research, the absolute dissolution amount of the carbonate rock test pieces for the two kinds of algae are shown in Table 1. The average of the absolute dissolution amount (ER) for *C. vulgaris* and *K. dissectum* was 0.02866 mg·(cm^2^·a)^−1^, compared to the control group (ST) which was 0.00997 mg·(cm^2^·a)^−1^. The difference of the ER values of the two kinds of algae was not statistically significant, while the difference between them and the control group were extremely significant (*p* < 0.01). Under the influences of the two kinds of algae, the dissolution of carbonate rock significantly increased (3 times of that of the control group), proving that algae function in the process of carbonate rock dissolution weathering.

**Table 1:** Absolute dissolution amount of carbonate test pieces.

Group	W_1_(g)	W_2_(g)	W_1_-W_2_(g)	ER (mg·(cm^2^·a)^−1^)	Mean of ER (mg·(cm^2^·a)^−1^)
CS①	0.5533	0.5525	0.0008	0.0299	
CS②	0.5375	0.5368	0.0007	0.0262	0.02866 ± 0.00125a
CS③	0.5802	0.5794	0.0008	0.0299	
KS①	0.4827	0.4820	0.0007	0.0262	
KS②	0.4854	0.4845	0.0009	0.0336	0.02866 ± 0.00249a
KS③	0.4808	0.4801	0.0007	0.0262	
ST①	0.4590	0.4588	0.0002	0.0075	
ST②	0.4579	0.4576	0.0003	0.0112	0.00997 ± 0.00125b
ST③	0.5005	0.5002	0.0003	0.0112	

W_1_, initial weight of test piece (g); W_2_, the weight of the test piece after placement (g); ER, annual absolute dissolution amount (mg·(cm^2^·a)^−1^); CS, *C. vulgaris* and carbonate rock slice group; KS, *K. dissectum* and carbonate rock slice group; CH, *C. vulgaris* group; KL, *K. dissectum* group. Different lowercase letters indicate significant differences (*p* < 0.01).

## 4 Discussion

### 4.1 Function and rule of the two kinds of algae on carbonate rock solutional weathering

The experimental algae used in this research were *Chlorella*, Chlorellaoideae, Charophyta and *Klebsormidium*, Klebsormidiophyceae, Charophyta. Both come from the same phylum, but are from a different family and genus. They showed dissolution effect on carbonate rock with a consistent pattern. Our results indicated that the life activities of the two kinds of algae increased the pH value of the culture solution. This is because the CO_2_ produced by the respiration of algae causes the reaction of “CO_2_ + H_2_O → HCO_3_^−^,” and HCO_3_^−^ causes pH to rise. Thus, the rise in pH cannot solely support the existence of dissolution process. This study does not completely agree with the point of view of Yu et al. (2004) that the occurance and degree of dissolution can be judged via the variation of pH value. Further analysis shows that CaCO_3_ is the main soluble chemical component of carbonate rocks, followed by CaMg(CO_3_)_2_. Adding carbonate rock slice into an algae culture solution will construct a theoretical reaction system: “CaCO_3_ + CO_2_ + H_2_O → Ca^2+^+2HCO_3_^−^, CaMg(CO_3_)_2_ + 2CO_2_ + 2H_2_O → Ca^2+^+Mg^2+^+4HCO_3_^−^.” The detection of Ca^2+^ and Mg^2+^ in the solution, combined with the variation of pH can fully support the occurrence of dissolution. In this work, we used a calcium-deficient medium, the significant Ca^2+^ rise detected in the solution, combined with the fact of pH increase with carbonate rock slices, sufficiently proved the dissolution effect of algae on carbonate rocks. Our results help to explain the paradox between the experimental outcomes and the theory proposed by Schwartz (1985) that the partial absorption and utilization of Ca^2+^ of algae makes the Ca^2+^ release, which is direct evidence of carbonate rock dissolution.

Mg^2+^ is a component of chlorophyll and an activator of enzymes (Beale, 1999). The algae culture medium usually contains a large amount of Mg^2+^, thus meeting the demand of Mg^2+^ in the process of chlorophyll synthesis. In this experiment, the culture medium contained a specific amount of Mg^2+^ (24 mg·L^−1^ ~ 25 mg·L^−1^). The decrease of Mg^2+^ reflects the absorption and utilization of algae. On the other hand, in the carbonate rock experiment groups, the content of Mg^2+^ increased after a period of decreasing, and surpassed that of the ST group, suggesting the occurrence of dissolution. The physiological activities of the algae are dynamic and quite complicated, the variation of Mg^2+^ in the culture medium may affect the utilization of Mg^2+^ by algae. Even though the Mg^2+^ content was extremely significantly correlated to pH, we still used the variation of Ca^2+^ concentration as a more important indicator of carbonate rock weathering and dissolution due to its much higher content in carbonate rocks. On this foundation, the rise in pH and the EC fluctuation can be used to indicate the velocity of dissolution.

Fe and Mn are necessary trace elements for algae growth and metabolism, which promote the proliferation of algae. Algae are sensitive to Fe^2+^. Through active or passive absorption, Fe^2+^ can be transported into algae cells (Kong et al., 2014). Mn is an essential element maintaining the structure of the chlorophyll membrane and is an involved in photolysis in the process of photosynthetic electron transfer (Rousch and Sommerfeld, 1999). In this study, the contents of Fe^2+^ and Mn^2+^ were negatively significantly correlated to AB and Chl-a (*p* < 0.05) (Figure 5), indicating the timely absorption and utilization of the two ions by algae. The Fe^2+^ and Mn^2+^ contents in algae-carbonate rock slices groups and pure algae groups were not significantly different. Fe^2+^ and Mn^2+^ are relatively insoluble, and the content was low in the test piece, which explains the continuous decrease of the concentration of the two ions, and also suggests that algae do not directly affect the dissolution of Fe^2+^and Mn^2+^. The contents of Fe^2+^ and Mn^2+^ decreased more in *C. vulgaris* than in *K. dissectum*, indicating the higher demand of Fe^2+^ and Mn^2+^ in *C. vulgaris*.

In sum, the dissolution effects of the two algae were manifested by the release of Ca^2+^and Mg^2+^, which present a similar pattern, that is, with the growth and metabolism of algae, the release of CO_2_, the synthesis and accumulation of EPS and CA, the dissolution of algae metabolites increases, and the release of Ca^2+^ and Mg^2+^ increases.

### 4.2 Carbonate rock solutional weathering mode of the two different ecotypes of algae

In this study, *C. vulgaris* and *K. dissectum* were isolated from the typical bare carbonate rock habitat. *C. vulgaris* is a single-cell green algae, which lives an aquatic or terrestrial life, and is distributed worldwide. *K. dissectum* is a multicellular filamentous green alga, which lives a terrestrial life, and is also widely distributed. These two algae from different ecotypes demonstrate different dissolution characteristics. In the current study, the EPS content was higher in the co-culture of algae and carbonate rock slices (Figure 5), suggesting that the carbonate rock slice promote EPS secretion in algae. Kang et al. (2008) demonstrated that exopolysaccharide layer increases with the alkalinity, which promotes the photosynthetic activity and improves the synthesis and accumulation of photosynthetic products. The secretion of exopolysaccharide could be a self-protection mechanism cell employed to adapt to high alkaline environment. In the current study, the alkalinity was higher in the co-cultures of algae and carbonate rock slices, which explains the enhanced EPS secretion, and also explains the enhanced EPS secretion during the early stages for *C. vulgaris* and the enhanced EPS secretion during the later stages for *K. dissectum* (Figure 2C). The EPS of *K. dissectum* was positively significantly correlated to contents of Ca^2+^ and Mg^2+^(Figure 5A), suggesting that the exopolysaccharide has an important carbonate rock dissolution function. The EPS secretion of *C. vulgaris* at early and middle stages were higher than that of the *K. dissectum*, although it did not significantly correlate to Ca^2+^ and Mg^2+^, reflecting the relatively weak dissolution ability of *C. vulgaris* EPS. These findings indicate two different survival strategies of algae. *C. vulgaris* is a spherical unicellular alga with a diameter of 5 μm, and usually lives in natural water. The first problem that needs to be overcome during the changing process from an aquatic to a terrestrial habitat is drought resistance. The colloid and water retention properties of EPS make it easier for algae to colonize and survive on bare rock. It is a critical strategy for *C. vulgaris* to secret more EPS at an early stage to ensure its survival on land. Dissolution is not its primary purpose. *K. dissectum* are typically terrestrial algae, with a multicellular filamentous structure, and are much more complicated than *C. vulgaris*. Compared to *C. vulgaris*, *K. dissectum* adapt to terrestrial life better. They can actively obtain inorganic ions from the living microenvironment through the chelation of metabolites such as EPS to meet their demands of life activities (Barker and Banfield, 1996). Whether there are differences between the EPS components of the two kinds of algae still needs further research.

Microorganisms in karst environment can express extracellular carbonic anhydrase (CA) (Wang et al., 2018), which is a metal enzyme with zinc as the active center. CA breaks the ionization balance of CaCO_3_ by catalyzing the hydration reaction of CO_2_, thus driving the dissolution of calcium carbonate. CA is commonly found in algae (Xie and Wu, 2014b). In this study, we detected CA activity in the culture solution of both algae, indicating that the two algae were capable of producing the enzyme. The correlation analysis showed that the CAA of *K. dissectum* positively correlated to contents of Ca^2+^ and Mg^2+^, although not significantly. The CAA of *C. vulgaris* positively significantly correlated to contents of Ca^2+^ and Mg^2+^, and the activity was even higher during early stages (Figure 5), suggesting that *C. vulgaris* carries on dissolution mainly through *CA*. *C. vulgaris* ensures its survival by secreting EPS, and obtains more inorganic ions from the environment through CAA. Therefore, different algae ecotypes have different ways of dissolving carbonate rocks due to their different survival strategies. Using the CAA level to indicate the strength of dissolution is only appropriate for those algae species which utilize the “CAA dissolution mode.” On the contrary, other algae may use another dissolution mode called “EPS dissolution mode,” which carries on the dissolution through EPS.

**Figure 6:**
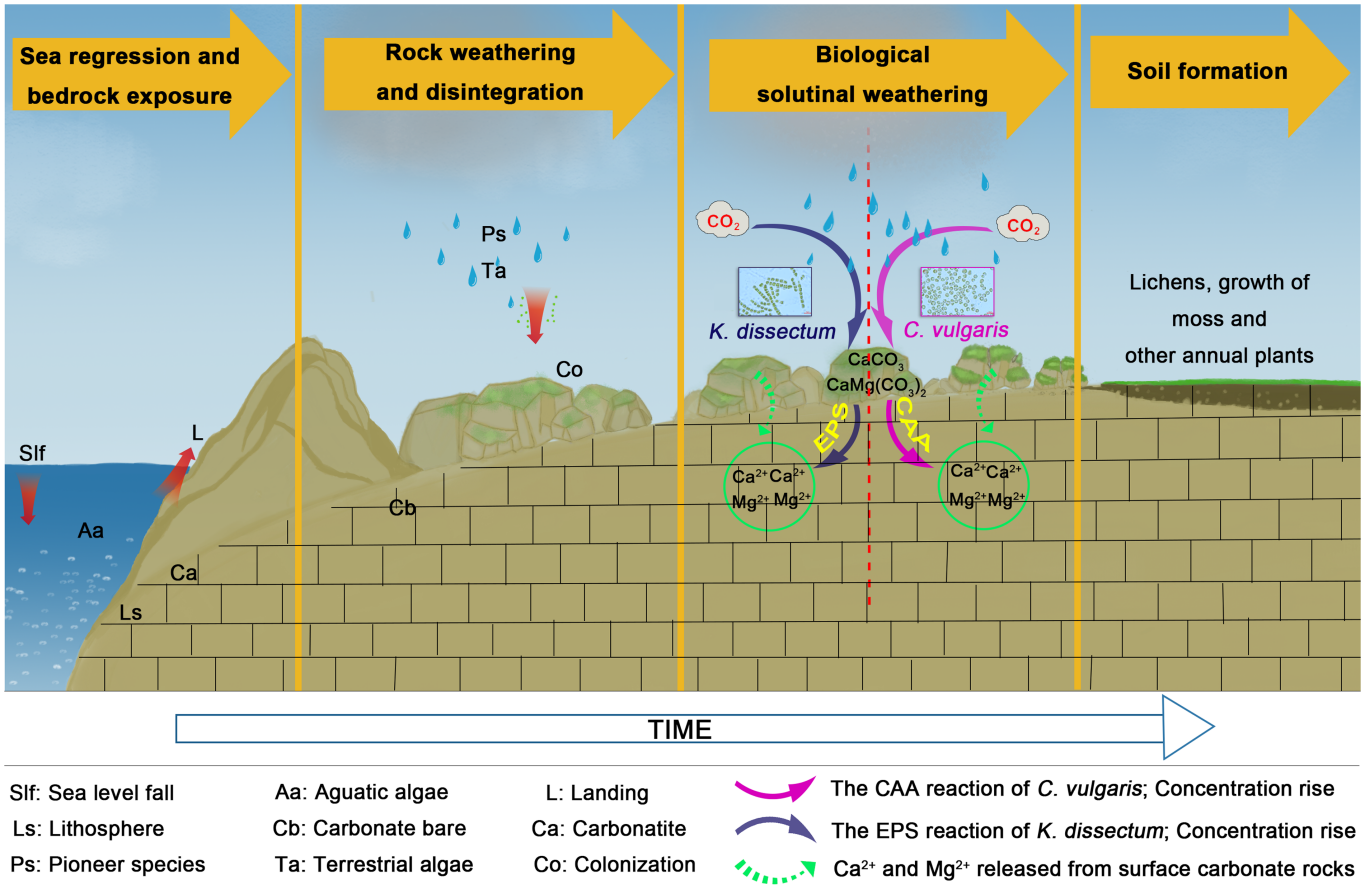
The dissolution mode of the *Klebsormidium* *dissectum* and *Chlorella vulgaris*.

In the present study, *C. vulgaris* and *K. dissectum* demonstrated the two different modes, respectively. Combing what we know about the different dissolution modes of these two species with our knowledge of their morphological characteristics and ecotypes, we proposed a hypothesis of dissolution mode selection for algae: aquatic-terrestrial unicellular algae adopt a CAA dissolution mode and terrestrial multicellular algae adopt an EPS dissolution mode (Figure 6). With the evolution from unicellular to multicellular, and aquatic to terrestrial, the dissolution mode of algae also changes from CAA to EPS. During the process of carbonate rocks weathering into soil, algae release CO_2_ through respiration. The catalytic effect of CA drives the reaction CO_2_ + H_2_O⇌HCO_3_^−^ + H^+^ moving to the right hand side, accelerating the production of carbonic acid, which reacts with the main minerals of carbonate rocks, CaCO_3_ and CaMg(CO_3_)_2_, resulting in the dissolution and release of Ca^2+^ and Mg^2+^. EPS produced by algae metabolism can be further oxidized into organic acids, which have equal or stronger dissolution abilities (Cao et al., 2001). The reaction can be summarized as:

$C.\;\mathrm{vulgaris}:CO_{2}+H_{2}O+\mathrm{CaMg}{\left(CO_{3}\right)}_{2}\overset{\;CAA\;}{\to }\;Ca^{2+}+Mg^{2+}+HC{O_{3}}^{-}$

$K.\;\mathrm{dissectum}:EPS\overset{\;O_{2}\;}{\to }\;R-\mathrm{COOH}$

$$\begin{array}{l} CO_{2}+H_{2}O+\mathrm{CaMg}{\left(CO_{3}\right)}_{2}+R-\mathrm{COOH}₂\\ \to Ca^{2+}+Mg^{2+}+HC{O_{3}}^{-} \end{array}$$

However, whether these two modes are ubiquitous still needs verification through repetitive research on more algae. Our results suggest that focusing on different algae ecotypes is necessary in the future research. On the one hand, it is critical to carry out in-depth research from the perspective of the systematic evolution of algae and the adaptive co-evolution of carbonate rocks in the terrestrial environment. On the other hand, setting up more scientifically accurate criteria for algae classification from the perspective of ecotype is also conducive to the algae taxonomy.

### 4.3 Establishment of pioneer status of algae in carbonate rock solutional weathering

Our quantitative study about *C. vulgaris* and *K. dissectum* in carbonate rock dissolution weathering suggests that algae play a pioneer role in the dissolution process. Under the simulated experiment conditions, the absolute dissolution amount of carbonate rocks with algae present was 0.02866 mg·cm^−2^·a^−1^, which was 3 times that of the solution without algae (Table 1). Although the two kinds of algae had different internal drives (“CAA dissolution mode” and “EPS dissolution mode”) for the dissolution, they had similar efficiency. For a specific alga, more accurate quantitative experiments need to be conducted to clarify what proportions of EPS and CAA contributed in the dissolution process. Roughly calculating, if the 540,000 km^2^ karst area in South China was completely covered by algae, the annual dissolution amount would be 154,800 tons. The value of dissolution and the soil-forming properties of algae cannot be ignored.

Scholars have been studying the weathering of carbonate rocks by bacteria since the 1930s (Paine et al., 1932). The studies were lately expanded to more microorganisms such as fungi (Schnabel, 1991 Kang et al., 2022). However, Pokrovsky et al. (2021) revealed that a negligible effect on Ca and Mg release from carbonates by bacteria and fungi. Zhang et al. (2017) calculated the absolute dissolution amount of the four mosses as 0.45–0.59 mg·(cm^2^·a)^−1^ through soil column leaching simulation experiments. However, the carbonate rock slices pieces used in this experimental system were not directly covered by mosses, but buried in soil and quartz sand, thus the obtained values were biased and may not accurately represent the pure dissolution amount of mosses. Comparing with moss plants, both algae and bacteria are small in size, yet the heterotrophic living style cannot actively bring CO_2_ into karst systems to accelerate dissolution through photosynthesis. The growth of moss plants on rock surfaces eroded by algae and lichens are generally recognized as the succession stage after algae. While accelerating the dissolution process, algae produce organic materials including EPS and CA, bring out Ca^2+^ and Mg^2+^ in rocks, absorb Fe^2+^ and Mn^2+^, and finally form soil through algal humification and the release of enriched elements. It is an important source of soil with certain structure and fertility. The soil thickness in the karst region is shallow, and the soil has limited carrying capacity for plants and storing nutrients (Zhang et al., 2021). In the case of neighboring soil, bare rock adorned with algae serves as a valuable nutrient reservoir. Therefore, algal plants play a pivotal role in the formation and swift accumulation of soils in karst regions. The results of this study suggest that algae are well-deserved pioneer organisms in the karst area, which assist carbonate dissolution and soil formation. As human activities continue to disrupt natural ecosystems, they are increasingly at risk. In karst regions, vegetation decline, soil erosion, and the loss of water and nutrients caused by rocky desertification are crucial ecological issues. However, there is currently a lack of solid or profound foundational research on the ecological processes of soil formation mechanisms, particularly in the karst regions. There is an urgent need to conduct quantitative research on the initial ecological processes of biogenic karst, which is the soil formation process, focusing on this critical issue. Through extensive field surveys and controlled laboratory experiments, terrestrial algal species can be identified that accelerate the biogenic karst process leading to accelerated karst rock desertification. This research aims to provide efficient and feasible measures for ecological restoration and management of karst rocky desertification in the karst regions.

## 5 Conclusion

In the process of weathering and soil-forming in the South China karst area, the two kinds of terrestrial green algae, spherical and filamentous, separated from the surface of carbonate rocks, have good dissolution ability with different dissolution characteristics. They promote the decomposition of carbonate rock minerals and the formation of original soil, revealing the pioneer role of algae in dissolution, which is reflected in:

(1) Through the rapid settlement and fast growth of algae, their synthesis and secretion of extracellular polysaccharides and carbonic anhydrase organic substances can accelerate the dissolution of carbonate rocks, providing important source of carbon.
(2) The dissolution of carbonate rocks by algae is mainly manifested in the release of calcium and magnesium ions and the enrichment of iron and manganese ions. The significant loss of calcium and magnesium ions causes the huge changes in volume and quality of rocks, and leads to the relative enrichment of iron and manganese oxides and clay minerals in weathering residual materials.
(3) The different dissolution modes of two algae reflect the coevolution of terrestrial algae and arid environments, as well as the adaptation of terrestrial algae in bare rock environments.

## Data availability statement

The original contributions presented in the study are included in the article/supplementary material, further inquiries can be directed to the corresponding author.

## Author contributions

NY: Writing – original draft, Writing – review & editing. JZ: Data curation, Formal analysis, Writing – review & editing. KX: Writing – original draft, Writing – review & editing. CY: Data curation, Formal analysis, Writing – review & editing. JL: Data curation, Formal analysis, Writing – review & editing. QC: Data curation, Formal analysis, Writing – review & editing.
